# Can Global Value Chain Participation Drive Green Upgrade in China’s Manufacturing Industry?

**DOI:** 10.3390/ijerph191912013

**Published:** 2022-09-22

**Authors:** Shi Wang, Hua Wang

**Affiliations:** 1 Institute of Area Studies, Xi’an International Studies University, Xi’an 710128, China; 2 “One Belt One Road” Economic and Trade Cooperation Innovation Team, Xi’an International Studies University, Xi’an 710128, China; 3 School of Economics and Finance, Xi’an International Studies University, Xi’an 710128, China; 4 School of Foreign Studies, Xi’an Jiaotong University, Xi’an 710049, China; 5 Center for Asia-Europe Studies, Xi’an Jiaotong University, Xi’an 710049, China

**Keywords:** global value chain participation, green upgrade of manufacturing industry, green technological progress

## Abstract

Engagement in the global division of labor has greatly influenced China’s economy and environment. With the multi-regional input–output (MRIO) framework, we calculate the global value chain (GVC) participation index of China’s 16 manufacturing sectors. We also measure the green upgrade index of manufacturing sectors based on the super-efficiency epsilon-based measure (SEBM) and the Malmquist–Luenberger (ML) index. In addition, the effect of GVC participation on the green upgrade of manufacturing sectors is empirically tested with a fixed effects regression model for panel data. Results show that: (1) sectors that rank high in the forward linkage-based GVC participation index also tend to rank high in the backward linkage-based GVC participation index; (2) the ML index is greater than 1 in most years, indicating that the green upgrade of China’s manufacturing sectors shows an uptrend; (3) for both forward and backward linkage, the rise of the GVC and complex GVC participation indexes significantly promotes the green upgrade of manufacturing sectors. Finally, GVC participation of China’s manufacturing sectors promotes green upgrade mainly through green technology progress. The conclusions have empirical evidence and policy implications for the advancement to medium- and high-end GVC participation and the green transition of China’s manufacturing sectors.

## 1. Introduction

With the progress of globalization, conventional industrial chains are unable to satisfy the professional division of labor in the world and embedding in the global value chain (GVC) gradually becomes the new solution for participation in the global division of labor. With the cost advantages from cheap labor, China actively engaged in the global division of labor. Consequently, China reaped great economic income, and became an indispensable part of the GVC. For developing countries including China, GVC participation helps improve manufacturing technologies and the industrialization level, thus promoting economic growth. However, developed countries and regions that dominate the GVC may transfer pollution-intensive production links to developing countries, causing serious environmental issues. With the transfer of pollution and technological blockade by developed countries, is it likely that China may turn into a “pollution haven” due to the global division of labor, thus affecting the green upgrade of its manufacturing sectors? What may be the mechanisms of such effects? Would the effects of different ways of GVC participation vary? Precise answers to these questions may have significant theoretical and practical implications for China’s manufacturing sectors’ ability to achieve value-chain climbing and green upgrade in the process of GVC embedding.

The contributions of this research are: first, we comprehensively and accurately measured the forward and backward linkage-based GVC participation of China’s manufacturing industry with the GVC participation index by Wang et al. [1], and calculated the green upgrade of China’s manufacturing industry with the super-efficiency epsilon-based measure (SEBM) and the Malmquist–Luenberger (ML) index; second, we empirically tested the effects and mechanisms of different GVC participation (forward and backward linkage) on the green upgrade of China’s manufacturing industry.

The results suggest that (1) sectors that rank high in the forward linkage-based GVC participation index also tend to rank high in the backward linkage-based GVC participation index, indicating the integration of different ways of GVC participation; (2) green total factor productivity, which indicates the green upgrade of China’s manufacturing sectors, shows an uptrend in the time period of this research; (3) for both forward and backward linkage, the rise of GVC and complex GVC participation indexes significantly promotes the green upgrade of manufacturing sectors; (4) the GVC participation of China’s manufacturing sectors promotes the green upgrade mainly through green technology progress.

The rest of the article is structured as follows: Section 2 reviews the key literature related to this research. Section 3 describes the methodology and data used in this article. Section 4 shows the calculation results of the indexes of the GVC participation and green upgrade of China’s manufacturing sectors. Section 5 empirically tests the effect and mechanism of GVC participation on the green upgrade of China’s manufacturing sectors. Finally, Section 6 concludes and proposes possible policy recommendations.

## 2. Literature Review

This study is related to three branches of the literature. The first is the measurement of the global value chain. The main calculation methods of the GVC include vertical specialization, upstreamness of production, and value-added deconstruction. Hummels et al. [2] suggested that a country or a branch of economy participated in vertical specialization through forward or backward linkage, therefore the proportion of forward and backward linkage in total exports could indicate the country’s level of participation in the GVC. Johnson and Noguera [3] defined value-added export as the implied domestic value added that was finally taken by foreign countries. Koopman et al. [4] relaxed the hypotheses in Hummels et al. [2] stepwise, and separated a country’s total export into domestic value added taken by foreign countries, the value added that returned to home country after export, value added outside the border, and double counting. They established indexes of GVC participation and location based on vertical specialization. Kee and Tang [5] proposed a concise framework to calculate the proportion of export by processing trade corporations in domestic value added, and applied the framework to China’s corporations to investigate the potential causes. With panel data of the world input–output database, Tian et al. [6] tested the effects of backward and forward linkage-based GVC participation on industrial upgrade. Using data from 35 sectors from 40 countries between 2000 and 2011, Pleticha [7] estimated the effect of GVC participation on the increased value in the production function framework.

The second branch of the literature is the measure of the green industrial upgrade. This article uses green total factor productivity (GTFP) to measure the green upgrade of manufacturing sectors. Chung et al. [8] considered pollution emissions as “bad” output and placed it in the total factor productivity measuring model to calculate the contribution of environment factors. Zofio and Prieto [9] calculated the environment efficiency, respectively, in two scenarios of pollutant disposal at will and pollutant weak disposability. Fan et al. [10] studied the overall and industry-specific environmentally sensitive productivity of Shanghai, China with the global Malmquist–Luenberger (GML) index model, which could overcome the issues of linear equations with no solution and lack of transitivity in the meta-frontier Malmquist–Luenberger (MML) model. Investigating the relationship between GTFP and political competition, Jin et al. [11] measured the GTFP of sample cities with stochastic frontier analysis. Mukherjee [12] studied the efficiency of energy utilization by manufacturing branches in the USA between 1970 and 2001 with the data envelopment analysis (DEA) model, with undesirable outputs. Among other previous studies on GTFP, Li et al. [13] used the non-radial and non-directional slacks-based measure of directional distance function and the Luenberger productivity index to measure the GTFP of China’s equipment manufacturing industry. Specifically, they constructed a panel threshold model to determine the impact of foreign direct investment (FDI) and trade openness on the GTFP of the equipment manufacturing industry. They found that FDI, export, human capital, and environmental regulation had significant positive effects, whereas import and technological innovation had significant negative effects. Based on the GTFP of 46 “Belt and Road” countries between 2004 and 2016, Qiu et al. [14] empirically tested the relationship between investment in innovation, quality of institutions, and GTFP. Liu et al. [15] used the direction distance function and the global Malmquist–Luenberger productivity index methods to measure green total factor productivity. Zhao et al. [16] measured GTFP in Chinese cities based on the Malmquist–Luenberger index of non-radial slacks-based measure of directional distance. On this basis, they analyzed the relationship between environmental regulation and GTFP in China, and the results showed that there were differences and nonlinearities among cities with different monitoring levels and different economic development levels. Other recent studies can be found in Song et al. [17] and Huang and Liu [18].

The third branch of the literature is the environmental effect of trade openness. A popular research topic in this field is the pollution haven hypothesis, i.e., that pollution-intensive industries from developed countries tend to be established in developing countries with relatively weak environmental regulations, thus creating pollution havens [19,20,21]. With the development of GVC research, scholars focused on the effect of GVC embedding on the environment. Liu et al. [22] suggested that the international specialization of production’s internal activities in the GVC considerably influenced developing countries. On one hand, the original equipment manufacturers of developing countries could benefit from technology and managerial expertise through GVC embedding. On the other hand, developing countries play a key role in global production in terms of energy- and carbon-intensive industries, and GVC embedding increases energy consumption and carbon dioxide emission, negatively affecting carbon emission efficiency [23]. With panel data of 17 manufacturing sectors between 2000 and 2014, Qu et al. [24] investigated the effect of GVC embedding on the green growth of China’s manufacturing sectors, from the perspectives of embedding location and embedding level. Dynamic panel results indicated that lifting the embedding location of the GVC could significantly promote the green growth of China’s manufacturing sectors. Using data of China’s manufacturing sectors between 2006 and 2015, Hu et al. [25] found that lifting the embedding location of the GVC significantly enhanced the efficiency of green technology innovation. Other recent studies included Liu et al. [26] and Meng and Zhao [27].

From the aforementioned papers, it can be seen that, first, previous studies often used the vertical specialization index proposed by Hummels et al. [2] or the method proposed by Koopman et al. [4] to calculate the level of GVC participation. However, the first method may double count the value added, whereas the second method only considered export and ignored the production that satisfied the domestic final demand. This article adopted the method of Wang et al. [1] to calculate the index of GVC participation, because this method can simultaneously solve the two issues and generate more complete and accurate results. Second, the extant literature seldom analyzed the environmental effect of different ways of GVC participation. This article tested the effects of different ways (forward and backward linkage-based) of GVC participation on the green upgrade of manufacturing sectors and, therefore, more persuasively revealed the relationship between GVC participation and the green upgrade of manufacturing sectors.

## 3. Methods

### 3.1. Calculation Method of GVC Participation

Hummels et al. [2] proposed the vertical specialization index to measure a country’s level of GVC embedding, whereas Koopman et al. [4] measured GVC participation by applying the value-added trade method to world input–output database. The first method needed stringent hypothetical conditions to estimate the level of GVC participation accurately and is not generally applicable to multiple country comparisons. The second method only considered export, but ignored the production that satisfied domestic final demand, therefore was unable to cover all the ways a country participates in GVC. Wang et al. [1] more comprehensively analyzed the ways a country participated in GVC, and established the indexes that simultaneously considered the ways and degrees of GVC participation to estimate the roles and contributions of a country or a department in global trade. By decomposing the directions of value added in domestic production and the sources of value added used in domestic production, they established the corresponding production decomposition model, which took domestic demand into consideration, therefore, more accurately demonstrated the roles of a country or a department in GVC. This article adopted the method of Wang et al. [1] and calculated the GVC participation of China’s manufacturing sectors based on multi-regional input–output framework. Specifically, this research measured GVC participation based on the value-added and final product decomposition method. According to Wang et al. [1], this article defined GVC participation based on forward and backward linkage as follows:

(1) $$\mathrm{GVC}\_f=\frac{V\_\mathrm{GVC}}{\mathrm{Va}}=\frac{V\_\mathrm{GVC}\_R}{\mathrm{Va}}+\frac{V\_\mathrm{GVC}\_D}{\mathrm{Va}}+\frac{V\_\mathrm{GVC}\_F}{\mathrm{Va}}$$

(2) $$\mathrm{GVC}\_b=\frac{Y\_\mathrm{GVC}}{Y}=\frac{Y\_\mathrm{GVC}\_R}{Y}+\frac{Y\_\mathrm{GVC}\_D}{Y}+\frac{Y\_\mathrm{GVC}\_F}{Y}$$

In the equations, GVC_f is the forward linkage-based GVC participation index, and GVC_b is the backward linkage-based GVC participation index. Va is the value added of a country or a certain branch. Y is the value added in the final product. V_GVC is the domestic value added implied in the intermediate product exports, and Y_GVC refers to the value added implied in intermediate product imports. V_GVC_R and Y_GVC_R is the value added implied in intermediate product exports that are directly taken by the country. In addition, V_GVC_D and Y_GVC_D represent the domestic value added that returns to and is taken by the country. V_GVC_F and Y_GVC_F refer to the value added that is indirectly taken by the country or re-exported to a third country. The higher a country’s forward linkage-based GVC participation is, the more likely it is that this country participates in GVC and division of labor by providing intermediate products. This country is located relatively high-end in GVC, playing the role of value exporter. Conversely, the higher a country’s backward linkage-based GVC participation is, the more likely that this country processes intermediate products imported from other countries. This country is located relatively low-end in GVC, plays the role of value importer.

Furthermore, based on forward and backward linkage, this article classified GVC participation as simple or complex, based on the circulation of intermediate products between countries. Specifically, simple GVC participation involves only one time of cross-border value adding trade, whereas complex GVC participation involves at least two times of cross-border value adding trade. Refer to the equations below:

(3) $$\mathrm{GVC}\_f\_s=\frac{V\_\mathrm{GVC}\_R}{\mathrm{Va}}$$

(4) $$\mathrm{GVC}\_f\_c=\frac{V\_\mathrm{GVC}\_D}{\mathrm{Va}}+\frac{V\_\mathrm{GVC}\_F}{\mathrm{Va}}$$

(5) $$\mathrm{GVC}\_b\_s=\frac{Y\_\mathrm{GVC}\_R}{Y}$$

(6) $$\mathrm{GVC}\_b\_c=\frac{Y\_\mathrm{GVC}\_D}{Y}+\frac{Y\_\mathrm{GVC}\_F}{Y}$$

Specifically, GVC_f_s is forward linkage-based simple GVC participation index, and GVC_f_c is forward linkage-based complex GVC participation index. GVC_b_s is backward linkage-based simple GVC participation index, and GVC_b_c is backward linkage-based complex GVC participation index.

This article investigated the sectors of China’s manufacturing sectors. Due to data availability and consistency of statistical methods, this article mainly adopted the sector standard of world input–output database (WIOD) and made necessary adjustments, i.e., combining WIOD’s manufacture of motor vehicles, trailers, and semi-trailers (c20) and manufacture of furniture, other manufacturing (c21) into c20-c21. The sectors in this research are described in Table 1.

### 3.2. Calculation Method of Green Upgrade of Manufacturing Sectors

According to Qu et al. [24], this article measured the green upgrade of China’s manufacturing sectors with green total factor productivity. The extant literature mainly used data envelopment analysis (DEA) model and slack-based measure (SBM) model to measure GTFP. The conventional radial DEA model can calculate the optimal distance, but is unable to distinguish the quality of the output. An improved version of the conventional DEA model, the SBM model takes slack variables into consideration and is able to distinguish the quality of the output. However, it uses the non-radial method to calculate the distance. An evolution from these two methods, the epsilon-based measure (EBM) proposed by Tone and Tsutsui [28], is a mixed distance functional model that combines all the merits of radial DEA and non-radial SBM models, therefore, can more accurately calculate the efficiency of decision-making unit (DMU). The main advantages of the EBM model are as follows: it can measure the ratio of improvement between the target value and actual value of DMU. It can also find the difference between the actual value and the target value by calculating the non-radial numerical values of inputs and outputs. The measuring results of EBM and conventional DEA suffer from the same limitations, i.e., the results of simultaneously effective DMUs are always 1, therefore, unable to distinguish the distances between effective DMUs. Compared to the conventional DEA model, the super-efficiency model solved the issue that multiple effective DMUs are unable to distinguish the values of efficiency. The main idea of super-efficiency model is to establish frontiers for all effective DMUs and obtain efficiency values that are usually larger than 1, thus distinguishing the effective DMUs. This research combined the super-efficiency model and EBM into SEBM to calculate the GTFP of China’s manufacturing industry with the expectation of solving the aforementioned issues. A brief introduction to the method is as follows: First, this article considered every manufacturing sector a DMU to establish production frontiers. Based on the EBM, and considering the issue of undesirable outputs, this article further expanded the model to the SEBM, based on undesirable outputs:

(7) $$\vec{D_{V}^{t}}\left(x_{k}^{t},y_{k}^{t},b_{k}^{t}\right)=min\frac{\theta -\varepsilon_{x}\sum_{i=1}^{m}\frac{\omega_{i}^{-}s_{i}^{-}}{x_{ik}}}{\varphi +\varepsilon_{y}\sum_{r=1}^{s}\frac{\omega_{r}^{+}s_{r}^{+}}{y_{rk}}+\varepsilon_{b}\sum_{p=1}^{q}\frac{\omega_{p}^{b-}s_{p}^{b-}}{b_{pk}}}$$

(8) $$s.t.\sum_{j=1}^{n}x_{ij}\lambda_{j}+s_{i}^{-}=\theta x_{ik},\;i=1,\cdots ,m$$

(9) $$\sum_{j=1}^{n}y_{rj}\lambda_{j}+s_{r}^{+}=\varphi y_{rk},\;r=1,\cdots ,s$$

(10) $$\sum_{j=1}^{n}b_{pj}\lambda_{j}+s_{p}^{b-}=\varphi b_{pk},\;p=1,\cdots ,q$$

(11) $$\lambda_{j}\geq 0,\;s_{i}^{-}\geq 0,\;s_{r}^{+}\geq 0,\;s_{p}^{b-}\geq 0$$

In the above equations, $\vec{D_{V}^{t}}$ is the directional distance function under the condition of variable returns to scale; λ is the linear combination of coefficients of decision-making units; θ is the programming parameter of the model’s radial component; ε is the parameter of the significance of the non-radial component part in the calculation of efficiency. $x_{ik}$, $y_{rk}$, and $b_{pk}$, respectively, indicate the *i*th input of the *k*th decision-making unit, the *r*th desirable output, and the *p*th undesirable output; $s_{i}^{-}$, $s_{r}^{+}$, and $s_{p}^{b-}$, respectively, indicate the slack variables of the *i*th input, the *r*th desirable output, and the *p*th undesirable output; $\omega_{i}^{-}$, $\omega_{r}^{+}$, and $\omega_{p}^{b-}$ indicate the relative weight of the *i*th input, the *r*th desirable output, and the *p*th undesirable output. In addition, $\sum_{i=1}^{m}\omega_{i}^{-}=1\;\left(\omega_{i}^{-}\geq 0\right)$.

The aforementioned SEBM model generates static values of efficiency, therefore, is unable to demonstrate the changes of the efficiency values over time. The index method can successfully solve this problem. By setting different index functions, the changes over time can be expressed directly with values. Therefore, this article adopted the frequently used ML index method to calculate the growth in GTFP of different manufacturing sectors and the dismantled items. Based on Equations (7)–(11), the ML index model is established from time period *t* to (*t* + 1) according to the directional distance function from the perspective of output:

(12) $$\;\mathrm{ML}\left(x^{t+1},y^{t+1},b^{t+1};x^{t},y^{t},b^{t}\right)={\left[\frac{\vec{D_{V}^{t}}\left(x^{t+1},y^{t+1},b^{t+1}\right)}{\vec{D_{V}^{t}}\left(x^{t},y^{t},b^{t}\right)}\times \frac{\vec{D_{V}^{t+1}}\left(x^{t+1},y^{t+1},b^{t+1}\right)}{\vec{D_{V}^{t+1}}\left(x^{t},y^{t},b^{t}\right)}\right]}^{\frac{1}{2}}\;\;\;\;\;\;\;\;\;=\frac{\vec{D_{V}^{t+1}}\left(x^{t+1},y^{t+1},b^{t+1}\right)}{\vec{D_{V}^{t}}\left(x^{t},y^{t},b^{t}\right)}\times {\left[\frac{\vec{D_{V}^{t}}\left(x^{t+1},y^{t+1},b^{t+1}\right)}{\vec{D_{V}^{t+1}}\left(x^{t+1},y^{t+1},b^{t+1}\right)}\times \frac{\vec{D_{V}^{t}}\left(x^{t},y^{t},b^{t}\right)}{\vec{D_{V}^{t+1}}\left(x^{t},y^{t},b^{t}\right)}\right]}^{1/2}\;\;=\mathrm{TC}\left(x^{t+1},y^{t+1},b^{t+1};x^{t},y^{t},b^{t}\right)\times \mathrm{EC}\left(x^{t+1},y^{t+1},b^{t+1};x^{t},y^{t},b^{t}\right)$$

In Equation (12), ML is the Malmquist–Luenberger index, and TC is the green technology change from period *t* to period (*t* + 1). EC is the green technology efficiency change from period *t* to period (*t* + 1). If ML, TC, or EC index is greater than 1, it means the sector’s GTFP, green technology progress, or green technology efficiency improves from period *t* to period (*t* + 1). Conversely, if these indexes are smaller than 1, it means the sector’s GTFP, green technology progress, or green technology efficiency deteriorates from period *t* to period (*t* + 1). Green technology progress and efficiency are the sources of GTFP growth. Green technology progress may expand the production possibility frontier thanks to major technology innovation or introduction from foreign countries. Green technology efficiency may improve because a favorable policy or political institutional reform improves factor allocation efficiency, thus pushing production closer to the frontier.

To preserve the consistency of the sample of GVC participation index, this article mainly measured the ML, EC, and TC of the 16 sectors of China’s manufacturing industry between 2000 and 2014. The explanation of the indicators of input and output are as follows.

(1) Labor input. Labor is an essential input for production, especially for the production and operation of manufacturing industry. This article uses the annual average number of all employees in the manufacturing sectors as labor input.

(2) Capital input. Economic growth theory suggests that capital input is one of the main factors of production. When calculating GTFP, materials are often considered the capital input. However, in statistical yearbooks, there are no ready-to-use data of capital stock, calling for estimates. This article used the perpetual inventory method [29]:

(13) $$K_{t}=I_{t}+\left(1-\alpha_{t}\right)K_{t-1}$$

In the equation, $K_{t}$ and $K_{t-1}$ is the capital stock of the current and previous time periods. $I_{t}$ is the investment of the current period, and $\alpha_{t}$ is the depreciation rate. This article used net value of a sector’s upscale fixed assets as baseline capital stock, and the difference of the original value of fixed assets as the investment of the current period. Variable depreciation rate was used. Calculations are as follows:

(14) $$\alpha_{t}=\frac{D_{t}}{Immobilisations_{t-1}}$$

(15) $$D_{t}=AD_{t}-AD_{t-1}$$

(16) $$AD_{t}=Immobilisations_{t}-NVFA_{t}$$

In the equations, $D_{t}$ is the depreciation in *t*th term. $AD_{t}$ and $AD_{t-1}$ are the accumulated depreciation of *t*th and (*t* − 1)th terms, respectively. $Immobilisations_{t}\;and\;Immobilisations_{t-1}$ is the original value of fixed assets in *t*th and (*t* − 1)th terms, respectively. $NVFA_{t}$ is the net value of fixed assets in the *t*th term.

(3) Energy input. As a key driving force of an economy, manufacturing industry is typically high in input and energy consumption. Therefore, energy is an essential factor input for production. However, GTFP includes not only low pollution but also low energy consumption. Therefore, it is necessary to consider energy input when measuring the GTFP of manufacturing industry. This article used total energy consumption as energy input.

(4) Desirable output. This article used industrial total value of output as desirable output. Because the current statistical data do not show the industrial total value of output between 2012 and 2014, this article used industrial sales value instead.

(5) Undesirable output. Different from the conventional total factor productivity (TFP), the calculation of GTFP requires the inclusion of pollution emission indicators. This article chose CO_2_ and SO_2_ as indicators of undesirable output. SO_2_ emission can be obtained directly from *China Statistical Yearbooks on Environment*, whereas CO_2_ emission data are not included. This article adopted the method provided in *2006 IPCC Guidelines for National Greenhouse Gas Inventories*, and substituted with carbon emission data of eight fossil fuels, namely, coal, charcoal, crude oil, fuel oil, gasoline, kerosene, diesel, and natural gas. Sector CO_2_ emission is obtained as follows:

(17) $$CO_{2}=\overset{8}{\underset{i=1}{\sum }}E_{i}\times NCV_{i}\times CEF_{i}\times COF_{i}\times \frac{44}{12}$$

In the equation, $CO_{2}$ is the carbon dioxide emission, and i=1,2,⋯,8, respectively, are the eight fossil fuels. $E_{i}$ is the original value of the *i*th fossil fuel consumption. $NCV_{i}$ is the average low calorific value of the *i*th fossil fuel. Data of the two parameters above were from *China Energy Statistical Yearbooks*. $CEF_{i}$ is the carbon emission coefficient of the *i*th fossil fuel, the data of which came from *2006 IPCC Guidelines for National Greenhouse Gas Inventories*. $COF_{i}$ is the carbon oxidation factor of the *i*th fossil fuel. This article sets all the values as 1, indicating complete oxidization. 44/12 is the ratio between the molecular masses of carbon dioxide and carbon.

### 3.3. Econometric Model Setting and Variable Explanations

To empirically test the effect of GVC participation on the green upgrade of China’s manufacturing industry, this article established the following econometric model:

(18) $$GTFP\_ML_{it}=\alpha_{0}+\alpha_{1}GVC_{it}+\alpha_{2}ES_{it}+\alpha_{3}EE_{it}+\alpha_{4}OS_{it}+\alpha_{5}FDI_{it}+\mu_{i}+\delta_{t}+\varepsilon_{it}$$

In the Equation (18), the dependent variable GTFP_ML is the GTFP calculated with the aforementioned SEBM model and ML index. It represents green upgrade of the manufacturing industry. GVC is the global value chain participation index of the manufacturing industry reported above. Therefore, parameter $\alpha_{1}$ measured the effect of GVC participation by China’s manufacturing industry on green upgrade. Other independent variables are all control variables. ES is the energy consumption structure, which is measured by the proportion of the sector’s coal consumption in total energy consumption. We do not consider other energy sources other than coal, because coal is the most important energy source in China’s energy consumption structure. Coal is a high-pollution, high-emission fuel, therefore, a decrease in coal consumption and an increase in other clean energy can significantly reduce pollution and emission, thus promoting GTFP. Therefore, consistent with Liu et al. [30] and Yu et al. [31], we use the proportion of coal consumption in the industry to measure the energy consumption structure. EE is energy efficiency, which was measured with the ratio between sector sales value and total energy consumption. OS is ownership structure, which was measured with the proportion of the total assets of state-owned industrial enterprises in total assets of all up-scale industrial enterprises. FDI is foreign direct investment, which was measured with the proportion of the sum of foreign and Hong Kong, Macau, and Taiwan capital in sector’s total paid-in capital. FDI influences GTFP mainly through scale effects of scale, structure, and technology. *i* is industry, and t is time. $\mu_{i}$ is the individual effect of the manufacturing sector. $\delta_{t}$ is the effect of time, and $\varepsilon_{it}$ is the random error.

The econometric regression model Equation (18) adopted in this paper is the panel data model. The advantage of panel data is that it can solve the problem of missing variables, can provide more information about the dynamic behavior of individuals, contains a large sample size, and the accuracy of estimation can be improved. The disadvantage is that the sample data usually do not satisfy the requirement of being independent and identically distributed, because the disturbance terms of the same individual in different periods generally have autocorrelation. Panel data are generally estimated by fixed effect model or random effect model. After the Hausman test, it is found that the probability value is less than the critical level of 0.05, which indicates that the null hypothesis is rejected, and the fixed effect model is significantly better than the random effect model. Therefore, this paper finally adopts panel data fixed effect model for regression analysis.

### 3.4. Data Resources

The calculation of GVC participation index of China’s manufacturing sectors is mainly based on the WIOD database. The latest published WIOD database is WIOD 2016 edition, which contains years from 2000 to 2014; therefore, the study of this paper is also limited to this period.

The fundamental data used to calculate green upgrade index of China’s manufacturing sectors came from *China Statistical Yearbooks, China Energy Statistical Yearbooks, China Statistical Yearbooks on Environment, China Industry Statistical Yearbooks*, and *2006 IPCC Guidelines for National Greenhouse Gas Inventories.* Data for econometric model were from *China Statistical Yearbooks, China Energy Statistical Yearbooks*, and *China Industry Statistical Yearbooks*. In addition, this article integrated sectors from the aforementioned yearbooks into the WIOD classification in Table 1.

## 4. Calculation Results and Analysis of GVC Participation and Green Upgrade of China’s Manufacturing Sectors

### 4.1. Overview of China’s Manufacturing Sectors

**Figure 1:**
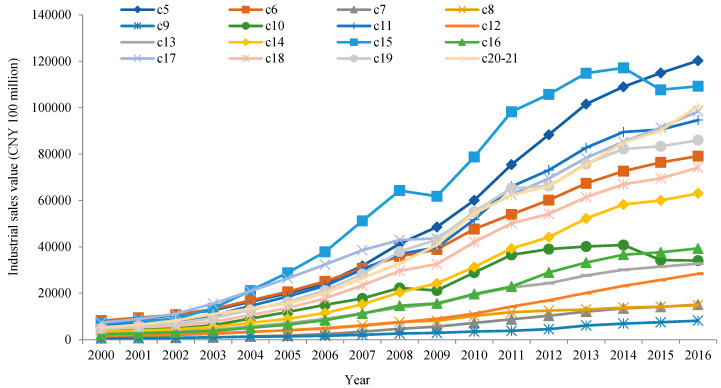
Industrial sales value of China’s manufacturing sectors between 2000 and 2016.

**Figure 2:**
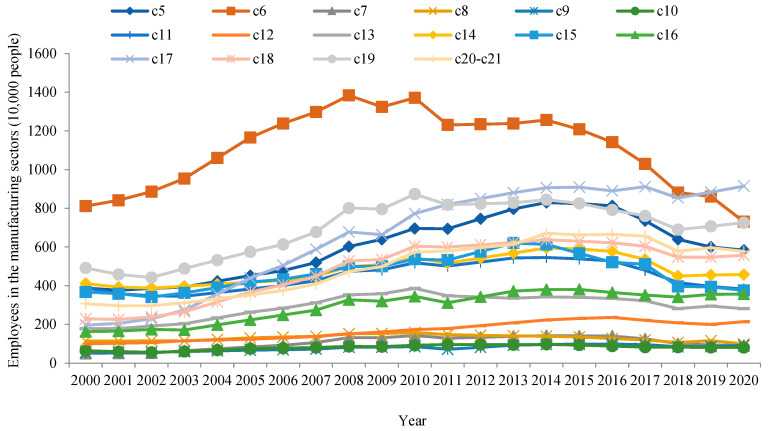
Number of employees in China’s manufacturing sectors between 2000 and 2020.

Before showing the calculation results of GVC participation and the green upgrade of China’s manufacturing sectors, we briefly introduce the essentials of China’s manufacturing sectors (see Figure 1 and Figure 2). The classification of sectors is adopted from Table 1 for consistency with the rest of the paper.

Figure 1 shows the industrial sales value of China’s manufacturing sectors between 2000 and 2016. In this paper’s main data sources for China’s manufacturing sectors, i.e., *China Statistical Yearbooks* and *China Industry Statistical Yearbooks*, the latest gross industrial output and value-added industrial output are only available up to 2011 and 2007, respectively, whereas industrial sales values are available up to 2016. Therefore, this paper adopted industrial sales value, which is very similar to gross industrial output, to represent the production of China’s manufacturing sectors. This method is also consistent with the aforementioned calculation of GTFP by sector. As Figure 1 shows, between 2000 and 2016, the 16 sectors’ industrial sales values generally increased, which is in accordance with China’s four decades of rapid economic growth since the reform and opening up. On average, the three sectors with the highest industrial sales value between 2000 and 2016 were: manufacture of basic metals; manufacture of food products, beverages, and tobacco products; and manufacture of computer, electronic, and optical products. In contrast, the three lowest sectors were, respectively: printing and reproduction of recorded media; manufacture of wood and products of wood and cork, except furniture, and manufacture of articles of straw and plaiting materials; and manufacture of paper and paper products.

Figure 2 shows the number of employees in China’s manufacturing sectors between 2000 and 2020. In this period, the numbers of employees of most sectors demonstrated the inverted U shape pattern with different inflection points. With reference to industrial sales values in Figure 1, it can be found that the labor productivity of China’s manufacturing industry increased. On average, the three sectors with the largest number of employees between 2000 and 2020 were: manufacture of textiles, clothing apparel, and leather products; manufacture of machinery and equipment not elsewhere classified; and manufacture of computer, electronic, and optical products. In contrast, the three sectors with the fewest number of employees were, respectively: printing and reproduction of recorded media; manufacture of coke and refined petroleum products; and manufacture of wood and products of wood and cork, except furniture, and manufacture of articles of straw and plaiting materials.

### 4.2. Calculation Results of GVC Participation of China’s Manufacturing Sectors

**Figure 3:**
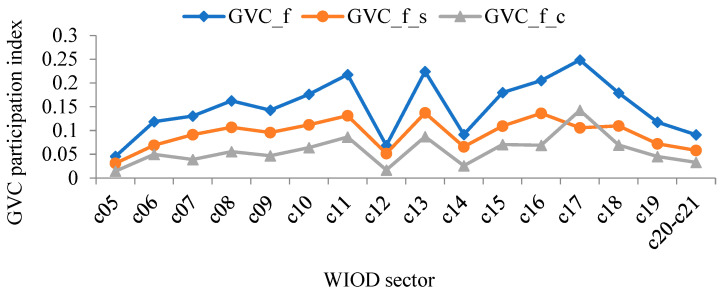
The forward linkage-based GVC participation index of China’s manufacturing sectors between 2000 and 2014 (annual average).

**Figure 4:**
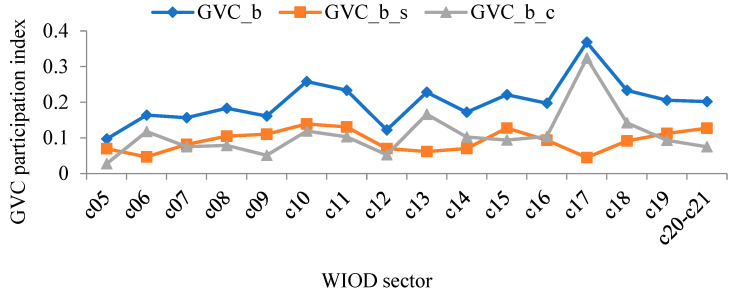
The backward linkage-based GVC participation index of China’s manufacturing sectors between 2000 and 2014 (annual average).

Using WIOD data and the aforementioned method, the level and composition of forward and backward linkage-based GVC participation of China’s manufacturing sectors were calculated. Results are shown in Figure 3 and Figure 4.

Comparing the different ways of participation in Figure 3 and Figure 4, it can be found that sectors that show higher forward linkage-based GVC participation tend to show higher backward linkage-based GVC participation. This result indicates that the ways that China’s manufacturing sectors participate in GVC are varied, i.e., China needs to play the role of both the supplier and the consumer. In addition, the two ways of participation are not contradictory: an optimized plan to participate in global trade helps coordinate the development of different sectors.

At the sector level, the three sectors with the highest forward linkage-based GVC participation index between 2000 and 2014 were: manufacture of computer, electronic, and optical products; manufacture of rubber and plastic products; and manufacture of chemicals and chemical products. In contrast, the three sectors with the lowest forward linkage-based GVC participation index were, respectively: manufacture of food products, beverages, and tobacco products; manufacture of basic pharmaceutical products and pharmaceutical preparations; and manufacture of motor vehicles, trailers, semi-trailers, and other transport equipment. The three sectors with the highest backward linkage-based GVC participation index between 2000 and 2014 were: manufacture of computer, electronic, and optical products; manufacture of coke and refined petroleum products; and manufacture of chemicals and chemical products. In contrast, the three sectors with the lowest backward linkage-based GVC participation index were, respectively: manufacture of food products, beverages, and tobacco products; printing and reproduction of recorded media; and manufacture of basic pharmaceutical products and pharmaceutical preparations. In summary, whether measuring forward or backward linkage-based GVC participation index, manufacture of computer, electronic and optical products was significantly higher than the other sectors. Manufacture of food products, beverages, and tobacco products was significantly lower than the other sectors.

Moreover, from the perspective of the composition of the GVC participation index, for both manufacture of computer, electronic, and optical products in Figure 3, and manufacture of textiles, clothing apparel, and leather products; manufacture of rubber and plastic products; manufacture of other non-metallic mineral products; manufacture of fabricated metal products, except machinery and equipment; manufacture of computer, electronic, and optical products; and manufacture of electrical equipment in Figure 4, the complex GVC participation index was higher than the simple GVC participation index, suggesting that when participating in the GVC, these sectors had multiple rounds of intermediate product circulation between countries and, therefore, were more deeply embedded in the GVC.

**Figure 5:**
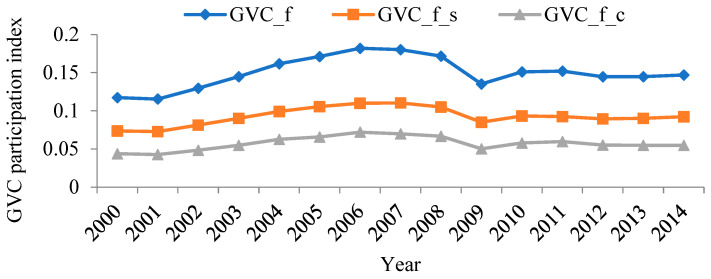
The forward linkage-based GVC participation index of China’s manufacturing sectors between 2000 and 2014.

**Figure 6:**
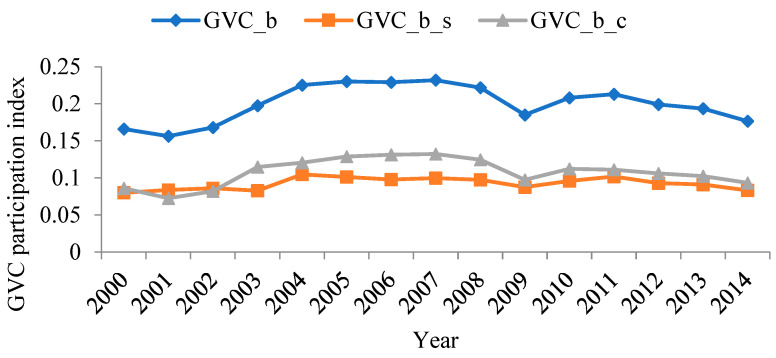
The backward linkage-based GVC participation index of China’s manufacturing sectors between 2000 and 2014.

Figure 5 and Figure 6 show the trends of the forward and backward linkage-based GVC participation index of China’s manufacturing sectors between 2000 and 2014. The evolution patterns of the forward and backward linkage-based GVC of China’s manufacturing sectors are basically consistent. Since joining the WTO in 2001, China’s GVC participation index also rose. It witnessed a dramatic downfall due to the 2008 global financial crisis. 2010 and 2014 were a “recovery period” for China’s manufacturing industry, and both forward and backward linkage-based GVC participation indexes rose again.

### 4.3. Calculation Results of Green Upgrade of China’s Manufacturing Sectors

**Figure 7:**
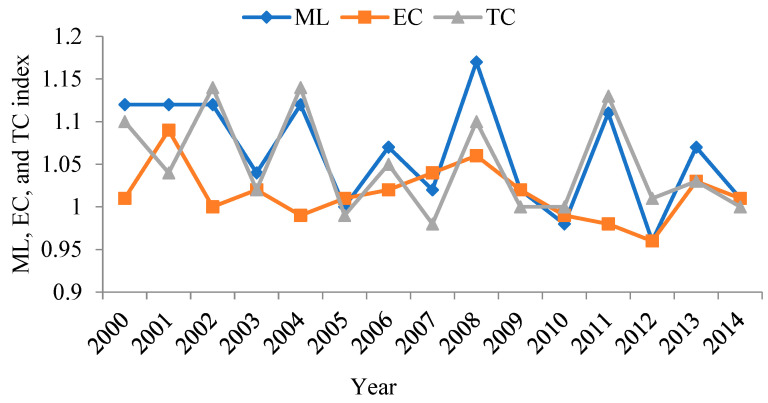
The GTFP of China’s manufacturing industry from 2000–2014: growth rate and disintegration.

The GTFP of China’s manufacturing industry can be obtained by entering data of the input–output indicator system into the SEBM model. Then, after calculating the growth rate of the GTFP of China’s manufacturing industry with the ML index, the ratios of distances between the technology level and production frontier of different time periods can generate the more reasonable growth rates as compared with the growth rates of simple GTFP in different time periods. Furthermore, the ML index can be deconstructed into green technology efficiency index (EC) and green technology progress index (TC). Calculation results are in shown Figure 7.

Figure 7 shows that the GTFP of China’s manufacturing industry was greater than 1 in most years except 2005, 2010, and 2012. The average value in the time range of the study is 1.063, indicating that the annual growth rate of the total GTFP of the manufacturing industry is 6.3%.

The dismantled items of ML, EC, and TC, respectively, generated average values of 1.015 and 1.048 in the time period of the study, indicating that the annual growth rates of green technology efficiency and green technology progress were 1.5% and 4.8%, respectively. This means that with the rising GTFP of China’s manufacturing industry, the contribution of green technology progress was relatively greater. Green technology progress has played a pivotal role in the green transition of the manufacturing industry, which can be attributed to China’s endeavor in technology development and independent innovation. In recent years, China introduced a massive amount of advanced equipment and techniques from abroad, and increased inputs in technology development and renovation, as well as the training of technicians, which all improved the renovation and independent innovation capacities of the enterprises.

It should be noted that ML, EC, and TC indicators all demonstrated a descending trend in the time period of the study. This indicates that, although the GTFP, green technology efficiency, and green technology progress of China’s manufacturing industry generally increased, the speed of increase gradually slowed down. In 2008, the value of ML was 1.17, the greatest growth rate in the time period of the study. The smallest growth rate was 0.96, in 2012. This was because in the earlier period, factor input could be allocated to different sectors reasonably under environmental regulations. After the financial crisis, many sectors tried to escape the predicament and reboot the economy at the cost of the environment.

**Figure 8:**
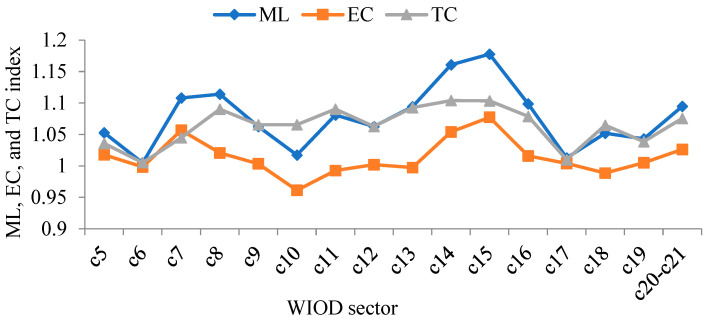
The GTFP of China’s manufacturing sectors from 2000–2014: growth rate and disintegration (annual average).

This article also calculated the ML, EC, and TC indicators of China’s manufacturing sectors between 2000 and 2014, as shown in Figure 8.

Specifically, sectors with ML, EC, and TC greater than 1 include: manufacture of food products, beverages and tobacco products; manufacture of wood and products of wood and cork, except furniture; manufacture of articles of straw and plaiting materials; manufacture of paper and paper products; printing and reproduction of recorded media; manufacture of basic pharmaceutical products and pharmaceutical preparations; manufacture of other non-metallic mineral products; manufacture of basic metals; manufacture of fabricated metal products, except machinery and equipment; manufacture of computer, electronic, and optical products; manufacture of machinery and equipment not elsewhere classified; and manufacture of motor vehicles, trailers, semi-trailers, and other transport equipment. Presumably, the GTFP, green technology efficiency, and green technology progress in these sectors were rising.

As mentioned above, the ML index and its dismantled items EC and TC are all year-on-year growth indexes. To obtain the corresponding absolute values, this article hypothesized that the GTFP of 1999 was 1, and values of the subsequent years were obtained with the index multiplication method and noted as GTFP_ML. The dismantled items were similarly processed and noted as GTFP_EC and GTFP_TC.

## 5. Empirical Results

### 5.1. Baseline Regression Results and Analyses

This article tests the effect of GVC participation of the manufacturing industry on the green upgrade from the perspective of different sectors, with data of 16 sectors from 2000 to 2014. The panel data fixed effect model was used. Because GVC participation can be classified into forward and backward linkage, regression results are shown in Table 2 and Table 3, respectively.

In Table 2, columns (1) and (2) show the effects of the forward linkage-based GVC participation index on the green upgrade of manufacturing industry. Columns (3) and (4) are effects of the forward linkage-based simple GVC participation index on the green upgrade of the manufacturing industry. Columns (5) and (6) are effects of the forward linkage-based complex GVC participation index on the green upgrade of the manufacturing industry. Columns (2), (4), and (6) include control variables. From Table 2, it can be seen that whether including or excluding control variables, the estimated coefficients of the forward linkage-based GVC participation index, simple GVC participation index, and the complex GVC participation index are quite similar, indicating the robustness of the regression results. This article mainly introduces the regression results of columns (2), (4), and (6) that include control variables. The coefficient of the forward linkage-based GVC participation index is positive and significant at a 1% level, indicating that the increase in the forward linkage-based GVC participation index significantly promoted the green upgrade of the manufacturing industry. The coefficient of the forward linkage-based simple GVC participation index is positive but statistically non-significant, indicating that the increase in the forward linkage-based simple GVC participation index did not promote the green upgrade of the manufacturing industry. The main reason could be that intermediate products that circulate only once between countries were mainly processing trade or second outsourcing. Consequently, inter- or intra-industry learning and exchange were constrained, preventing technology spillover. The coefficient of the forward linkage-based complex GVC participation index is positive and significant at a 1% level, indicating that the increase in the forward linkage-based complex GVC participation index significantly promoted the green upgrade of the manufacturing industry. In the process of GVC embedding, intermediate products that circulated multiple times among countries cast a greater influence: corporations obtained more opportunities to learn and obtain technology spillover, promoting GTFP and a green upgrade.

In Table 3, columns (7) and (8) show the effects of the backward linkage-based GVC participation index on the green upgrade of the manufacturing industry. Columns (9) and (10) are effects of the backward linkage-based simple GVC participation index on the green upgrade of the manufacturing industry. Columns (11) and (12) are the effects of the backward linkage-based complex GVC participation index on the green upgrade of the manufacturing industry. Columns (8), (10), and (12) include control variables. From Table 3, it can be seen that whether including or excluding control variables, the estimated coefficients of the backward linkage-based GVC participation index, simple GVC participation index, and complex GVC participation index are quite similar, indicating the robustness of the regression results. This article mainly introduces the regression results of columns (8), (10), and (12) that include control variables. The coefficient of the backward linkage-based GVC participation index is positive and significant at a 1% level, indicating that the increase in the backward linkage-based GVC participation index significantly promoted the green upgrade of the manufacturing industry. The coefficient of the backward linkage-based simple GVC participation index is positive but statistically non-significant, a result that was similar with that of the forward linkage-based simple GVC participation index: the increase in the backward linkage-based simple GVC participation index did not promote the green upgrade of the manufacturing industry. The coefficient of the backward linkage-based complex GVC participation index is positive and significant at a 1% level, indicating that the increase in the backward linkage-based complex GVC participation index significantly promoted the green upgrade of the manufacturing industry. In the process of circulating multiple times among countries, intermediate products were mainly used for the production of components and parts of higher technology level, leading to more technology spillover and promoting GTFP of the manufacturing industry.

Comparing column (2) in Table 2 and column (8) in Table 3, it can be found that the increase in the forward linkage-based GVC participation index has a greater promoting effect on the green upgrade of the manufacturing industry, which is consistent with the finding of Koopman et al. [4]. Compared with the backward linkage-based GVC, the forward linkage-based GVC can obtain more value added and cause less pollution, thus more significantly promotes a green upgrade.

As for control variables, in Table 2 and Table 3 the coefficients of energy consumption structure (ES) are negative and significant at a 10% level, indicating the increase of coal in energy consumption may impede a green upgrade of China’s manufacturing industry. Since the reform and opening up, China’s coal-dominated energy consumption kept soaring in an extensive economic development mode. The carbon emission coefficient of coal ranks first among fossil fuels; therefore, it harms the environment the most and impedes the GTFP of the manufacturing industry. The coefficients of energy efficiency are positive and significant at a 10% level, indicating that an increase in energy efficiency leads to a significant improvement in the GTFP of the manufacturing industry. Ownership structure had no significant effect on the green upgrade of the manufacturing industry in all models, indicating that, although state-owned enterprises had an advantage in scale of economy, they lacked in motivation for technology innovation. This situation seriously impeded the improvement of the production efficiency and GTFP of the manufacturing industry. The coefficients of FDI were all negative and significant at a 5% level, indicating that an increase in FDI involvement restricted the green upgrade of the manufacturing industry. This seems to support the renowned pollution haven hypothesis (Walter and Ugelow [32]). This hypothesis suggests that the high-pollution enterprises in developed countries may not meet the increasingly stringent environmental regulation requirements, or use high-pollution production technologies in developing countries to dramatically drive down the cost. Consequently, high-pollution enterprises enter into developing countries with lower environmental regulation levels via FDI, causing pollution in their production processes. At the same time, to stimulate rapid economic growth, China spontaneously attracted large amounts of FDI with favorable policies of taxation and land. However, such FDI are often the high-pollution production links that developed countries transferred to China, thus may increase pollution and emission, therefore reduce the GTFP of China’s manufacturing industry.

### 5.2. Robustness Analysis

To ensure the robustness of the conclusions, this study adopted the GVC participation index constructed in Koopman et al. [4] to test the baseline regression results. The calculation of the index is as follows:

(19) $$GVC\_Koopman=\frac{IV}{Ex}+\frac{FVA}{Ex}$$

In the equation, GVC_Koopman is the GVC participation index in Koopman et al. [4]. IV is the indirect value added of exports. FVA is foreign value added in exports. Ex is export value.

Based on Koopman et al. [4], Swarnali et al. [33] further distinguished the forward and backward linkage of GVC:

(20) $$GVC\_Koopman\_f=\frac{IV}{Ex}$$

(21) $$GVC\_Koopman\_b=\frac{FVA}{Ex}$$

GVC_Koopman_f and GVC_Koopman_b are forward and backward linkage-based GVC participation indexes calculated by Koopman et al.’s [4] method, respectively. GVC_Koopman_f is the proportion of a certain sector of a country’s intermediate inputs in other countries’ exports. GVC_Koopman_b is the proportion of value from other countries in a certain sector of a country’s exports. It should be noted that the method of Koopman et al. [4] did not distinguish simple and complex GVC participation.

To keep the results comparable to baseline regression, we only tested the effects of GVC_Koopman_f and GVC_Koopman_b on the green upgrade of the manufacturing industry. Regression results are in Table 4.

In Table 4, columns (13) and (14) demonstrate the effects of GVC_Koopman_f and GVC_Koopman_b on the green upgrade of the manufacturing industry, respectively. It can be found that both coefficients are positive and statistically significant at a 5% level. In addition, the results are similar to column (2) in Table 2 and column (8) in Table 3, respectively, indicating that the baseline regression results are robust.

### 5.3. Mechanism Analyses

As mentioned above, the indicator of the green total factor productivity (GTFP_ML) can be separated into green technology efficiency (GTFP_EC) and green technology progress (GTFP_TC). Therefore, this article investigated the mechanism of GVC participation promoting the green upgrade of the manufacturing industry by testing the effect of GVC participation on GTFP_EC and GTFP_TC. Regression results are shown in Table 5.

In Table 5, columns (15) and (16), respectively, show the effects of forward and backward linkage-based GVC participation on GTFP_EC. Columns (17) and (18), respectively, show the effects of forward and backward linkage-based GVC participation on GTFP_TC. It can be seen that, whether considering forward or backward GVC participation, the regression coefficients of GTFP_TC are positive and significant at a 5% level, indicating that GVC participation significantly promoted the green upgrade of the manufacturing industry through green technology improvement. The regression coefficients of GTFP_EC are positive but statistically non-significant, suggesting that in the process of GVC participation and division of labor, China’s manufacturing enterprises may have been in contact with the advanced institutions and administration systems of developed countries and learning green production management experiences. However, due to factors at the institutional level, these enterprises were inefficient in the adoption of these practices. Simultaneously, developed countries transferred low value-adding production links and strictly controlled the spillover of core management ideas, which eventually impeded GVC participation from promoting the green upgrade of manufacturing industry through green technology efficiency. The above analysis indicates that the GVC participation of China’s manufacturing industry promotes green upgrade mainly through green technology progress.

## 6. Conclusions

With the multi-regional input–output framework, this article calculates the GVC participation index of China’s manufacturing sectors and measures the green upgrade index of manufacturing sectors based on super-efficiency epsilon-based measure and the Malmquist–Luenberger index. Based on this, we analyze the impact of GVC participation on the green upgrade of the manufacturing industry using the panel data fixed effect model. Results show that: (1) at the sector level, sectors that rank high in the forward linkage-based GVC participation index also tend to rank high in the backward linkage-based GVC participation index, indicating the integration of different ways of GVC participation and coordinated development of upstream and downstream industries; (2) the ML index is greater than 1 in most years, indicating that green total factor productivity shows an uptrend in the time period of this research; (3) for both forward and backward linkage, a rise of the GVC and the complex GVC participation indexes significantly promotes the green upgrade of manufacturing sectors, whereas a rise of simple GVC participation index did not promote green upgrade; (4) the GVC participation of China’s manufacturing sectors promotes green upgrade mainly through green technology progress. The academic contribution of this research is that it accurately measured the participation in the GVC division of labor by China’s manufacturing industry and, stemming from that, this research identified the effect of GVC participation by China’s manufacturing industry on green upgrade. This renders theoretical significance for promoting the green transition of China’s open economy.

## 7. Policy Recommendations

Based on the above conclusions, this article proposes the following policy recommendations. In terms of GVC embedding, differentiated promotion policies should be practiced according to the characteristics and the GVC positions of the sectors. For producer-driven sectors, the production and processing length of the value chain should be increased to enhance the level of processing. Trade openness and value-adding capacity should be further promoted, and the outflow of ecological factors should be reduced. China should strive to eliminate the dependence on low-end, high energy-consumption heavy industries, and accelerate the embedding in high-end links, such as research and development and sales service. Only in this way can China can break through the “low-end lock-in” by the developed countries. For buyer-driven sectors, China should try to explore its market potential and enhance market controllability, thereby making the backward linkage of the value chain possible, and the enterprises can climb up the GVC by sufficiently benefiting from the advantages of China’s domestic market. In terms of green development, China should improve its environment regulations for high-pollution and high-emission sectors, and emphasize the ecological costs in production processes, thus raising the standards for FDI entrance and improving FDI quality. In addition, clean production technology should be particularly introduced to improve resource utilization efficiency and clean production. Backward production capacity should be eliminated, and clean, green industrial development should be emphasized more.

## Figures and Tables

**Table 1:** Sector descriptions.

WIOD Code (2016 Version)	WIOD Sector Description
c05	Manufacture of food products, beverages, and tobacco products
c06	Manufacture of textiles, clothing apparel, and leather products
c07	Manufacture of wood and products of wood and cork, except furniture; manufacture of articles of straw and plaiting materials
c08	Manufacture of paper and paper products
c09	Printing and reproduction of recorded media
c10	Manufacture of coke and refined petroleum products
c11	Manufacture of chemicals and chemical products
c12	Manufacture of basic pharmaceutical products and pharmaceutical preparations
c13	Manufacture of rubber and plastic products
c14	Manufacture of other non-metallic mineral products
c15	Manufacture of basic metals
c16	Manufacture of fabricated metal products, except machinery and equipment
c17	Manufacture of computer, electronic, and optical products
c18	Manufacture of electrical equipment
c19	Manufacture of machinery and equipment not elsewhere classified
c20-c21	Manufacture of motor vehicles, trailers, semi-trailers, and other transport equipment

**Table 2:** Baseline regression results (forward linkage-based GVC participation).

Variables	GTFP_ML					
	(1)	(2)	(3)	(4)	(5)	(6)
GVC_f	0.32 ** (0.13)					
GVC_f		0.36 *** (0.14)				
GVC_f_s			0.15 (0.03)			
GVC_f_s				0.17 (0.07)		
GVC_f_c					0.77 *** (0.13)	
GVC_f_c						0.83 *** (0.15)
ES		−2.78 ** (0.18)		−2.13 * (0.09)		−2.79 ** (0.18)
EE		0.02 * (0.01)		0.01 * (0.01)		0.01 * (0.01)
OS		−0.01 (0.02)		0.02 (0.03)		0.01 (0.01)
FDI		−1.13 *** (0.54)		−1.21 *** (0.62)		−1.17 ** (0.56)
Constant	0.95 * (0.07)	0.91 ** (0.07)	0.86 * (0.07)	0.81 * (0.06)	0.22 ** (0.05)	0.31 ** (0.06)
Industry Fixed Effect	Yes	Yes	Yes	Yes	Yes	Yes
Year Fixed Effect	Yes	Yes	Yes	Yes	Yes	Yes

Note: Robust standard errors are in parentheses. ***, **, and * indicate significant at 1%, 5%, and 10% levels, respectively.

**Table 3:** Baseline regression results (backward linkage-based GVC participation).

Variables	GTFP_ML					
	(7)	(8)	(9)	(10)	(11)	(12)
GVC_b	0.27 ** (0.15)					
GVC_b		0.30 *** (0.15)				
GVC_b_s			0.07 (0.01)			
GVC_b_s				0.11 (0.03)		
GVC_b_c					0.61 *** (0.09)	
GVC_b_c						0.72 *** (0.18)
ES		−3.77 ** (0.17)		−4.18 ** (0.16)		−3.62 ** (0.16)
EE		0.04 * (0.02)		0.02 * (0.01)		0.01 * (0.01)
OS		0.03 (0.02)		0.02 (0.01)		0.02 (0.01)
FDI		−1.22 *** (0.68)		−1.19 *** (0.51)		−1.34 *** (0.64)
Constant	0.57 ** (0.04)	0.49 ** (0.01)	0.66 ** (0.04)	0.73 ** (0.01)	0.15 (0.01)	0.38 (0.01)
Industry Fixed Effect	Yes	Yes	Yes	Yes	Yes	Yes
Year Fixed Effect	Yes	Yes	Yes	Yes	Yes	Yes

Note: Robust standard errors are in parentheses. ***, **, and * indicate significant at 1%, 5%, and 10% levels, respectively.

**Table 4:** Robustness test results.

Variables	GTFP_ML	
	(13)	(14)
GVC_Koopman_f	0.23 ** (0.17)	
GVC_Koopman _b		0.25 ** (0.39)
Control Variables	Yes	Yes
Industry Fixed Effect	Yes	Yes
Year Fixed Effect	Yes	Yes

Note: Robust standard errors are in parentheses. ** indicates significant at 5% level.

**Table 5:** Regression results of mechanism analyses.

Variables	GTFP_EC		GTFP_TC	
	(15)	(16)	(17)	(18)
GVC_f	0.02 (0.01)		0.51 *** (0.08)	
GVC_b		0.11 (0.03)		0.46 ** (0.05)
Control Variables	Yes	Yes	Yes	Yes
Industry Fixed Effect	Yes	Yes	Yes	Yes
Year Fixed Effect	Yes	Yes	Yes	Yes

Note: Robust standard errors are in parentheses. *** and ** indicate significant at 1% and 5% levels, respectively.

## Data Availability

The data presented in this study are available on request from the corresponding author.

## References

[1] Wang Z., Wei S.-J., Yu X., Zhu K. (2017). Measures of Participation in Global Value Chains and Global Business Cycles.

[2] Hummels D., Ishii J., Yi K.-M. (2001). The nature and growth of vertical specialization in world trade. J. Int. Econ..

[3] Johnson R.C., Noguera G. (2012). Accounting for intermediates: Production sharing and trade in value added. J. Int. Econ..

[4] Koopman R., Wang Z., Wei S.-J. (2014). Tracing value-added and double counting in gross exports. Am. Econ. Rev..

[5] Kee H.L., Tang H. (2016). Domestic value added in exports: Theory and firm evidence from China. Am. Econ. Rev..

[6] Tian K., Dietzenbacher E., Jong-A-Pin R. (2022). Global value chain participation and its impact on industrial upgrading. World Econ..

[7] Pleticha P. (2021). Who benefits from global value chain participation? Does functional specialization matter?. Struct. Chang. Econ. Dyn..

[8] Chung Y.H., Färe R., Grosskopf S. (1997). Productivity and undesirable outputs: A directional distance function approach. J. Environ. Manag..

[9] Zofío J.L., Prieto A.M. (2001). Environmental efficiency and regulatory standards: The case of CO_2_ emissions from OECD industries. Resour. Energy Econ..

[10] Fan M., Shao S., Yang L. (2015). Combining global Malmquist–Luenberger index and generalized method of moments to investigate industrial total factor CO_2_ emission performance: A case of Shanghai (China). Energy Policy.

[11] Jin G., Shen K., Li J. (2020). Interjurisdiction political competition and green total factor productivity in China: An inverted-U relationship. China Econ. Rev..

[12] Mukherjee K. (2008). Energy use efficiency in US manufacturing: A nonparametric analysis. Energy Economics.

[13] Li Y., Wu Y., Chen Y., Huang Q. (2021). The influence of foreign direct investment and trade opening on green total factor productivity in the equipment manufacturing industry. Appl. Econ..

[14] Qiu W., Zhang J., Wu H., Irfan M., Ahmad M. (2022). The role of innovation investment and institutional quality on green total factor productivity: Evidence from 46 countries along the “Belt and Road”. Environ. Sci. Pollut. Res..

[15] Liu Y., Yang Y., Li H., Zhong K. (2022). Digital economy development, industrial structure upgrading and green total factor productivity: Empirical evidence from China’s cities. Int. J. Environ. Res. Public Health.

[16] Zhao M., Liu F., Sun W., Tao X. (2020). The relationship between environmental regulation and green total factor productivity in China: An empirical study based on the panel data of 177 cities. Int. J. Environ. Res. Public Health.

[17] Song Y., Hao F., Hao X., Gozgor G. (2021). Economic policy uncertainty, outward foreign direct investments, and green total factor productivity: Evidence from firm-level data in China. Sustainability.

[18] Huang Q., Liu M. (2022). Trade openness and green total factor productivity: Testing the role of environment regulation based on dynamic panel threshold model. Environ. Dev. Sustain..

[19] Cole M.A. (2004). Trade, the pollution haven hypothesis and the environmental Kuznets curve: Examining the linkages. Ecol. Econ..

[20] López L.A., Arce G., Kronenberg T., Rodrigues J.F. (2018). Trade from resource-rich countries avoids the existence of a global pollution haven hypothesis. J. Clean. Prod..

[21] Cohen G., Jalles J.T., Loungani P., Marto R., Wang G. (2019). Decoupling of emissions and GDP: Evidence from aggregate and provincial Chinese data. Energy Econ..

[22] Liu H., Li J., Long H., Li Z., Le C. (2018). Promoting energy and environmental efficiency within a positive feedback loop: Insights from global value chain. Energy Policy.

[23] Li J., Lin B. (2017). Does energy and CO_2_ emissions performance of China benefit from regional integration?. Energy Policy.

[24] Qu C., Shao J., Cheng Z. (2020). Can embedding in global value chain drive green growth in China’s manufacturing industry?. J. Clean. Prod..

[25] Hu D., Jiao J., Tang Y., Han X., Sun H. (2021). The effect of global value chain position on green technology innovation efficiency: From the perspective of environmental regulation. Ecol. Indic..

[26] Liu H., Zong Z., Hynes K., De Bruyne K. (2020). Can China reduce the carbon emissions of its manufacturing exports by moving up the global value chain?. Res. Int. Bus. Financ..

[27] Meng F., Zhao Y. (2022). How does digital economy affect green total factor productivity at the industry level in China: From a perspective of global value chain. Environ. Sci. Pollut. Res..

[28] Tone K., Tsutsui M. (2010). An epsilon-based measure of efficiency in DEA–a third pole of technical efficiency. Eur. J. Oper. Res..

[29] Christensen L.R., Jorgenson D.W. (1969). The measurement of US real capital input, 1929–1967. Rev. Income Wealth.

[30] Liu Y., Zhao G., Zhao Y. (2016). An analysis of Chinese provincial carbon dioxide emission efficiencies based on energy consumption structure. Energy Policy.

[31] Yu S., Zheng S., Li X. (2018). The achievement of the carbon emissions peak in China: The role of energy consumption structure optimization. Energy Econ..

[32] Walter I., Ugelow J.L. (1979). Environmental policies in developing countries. Ambio.

[33] Swarnali A., Maximiliano A., Ruta M. (2015). Depreciations without Exports? Global Value Chains and the Exchange Rate Elasticity of Exports.

